# Anxious Dads and Depressed Moms: Child Disability and the Mental Health of Parents

**DOI:** 10.1002/hec.4962

**Published:** 2025-04-10

**Authors:** Derek Asuman, Tinna Laufey Ásgeirsdóttir, Johan Jarl

**Affiliations:** ^1^ Department of Public Health Aarhus University Aarhus Denmark; ^2^ Department of Economics University of Iceland Reykjavik Iceland; ^3^ Health Economics Department of Clinical Sciences (Malmo¨) Lund University Lund Sweden

**Keywords:** anxiety, cerebral palsy, children, depression, disability, event study, mental health, parental penalty, sleeping disorders, Sweden

## Abstract

Having a child with a disability undoubtedly affects parents in many ways, including their well‐being. However, the specific mental health trajectories of parents, differentiated by the severity of impairments and parental roles, remain under‐explored. We investigate the mental‐health effects of having a child with a disability. Using individual‐level register data from Sweden, we exploit the epidemiological features of Cerebral Palsy (CP) to estimate causal effects. Results show that prescriptions for mental‐health disorders increase after the birth of a child with CP. While fathers are more likely to be dispensed anti‐anxiety medications, dispensed medications for anti‐depressants increase for mothers. Further, the effects are larger for parents of children with severe impairments but do not differ across parental characteristics. Our findings highlight the need for support and assistance for families with children with disabilities.

## 1 Introduction

A large body of literature has reported that the transition to parenthood affects a broad spectrum of outcomes, albeit differently for fathers and mothers. Mothers, for example, face worse labor‐market outcomes (Kleven et al. 2019; Rabaté and Rellstab 2021; Cortés and Pan 2023; Casarico and Lattanzio 2023; Lebedinski et al. 2023) and poorer mental‐health (Ahammer et al. 2023; Hart et al. 2023; Dehos et al. 2024) after the birth of a child compared to fathers. These child penalties, it is argued, arise from parental differences in time spent on child‐related non‐market activities, with mothers being the primary caregivers to young children (Guryan et al. 2008; Borra et al. 2021; Cheung et al. 2025). The care demands of parenting differ greatly between parents of children with disabilities and typically developing children, highlighting the need to consider how a child's disability can impact parental roles and outcomes.

About 6% of children born each year are diagnosed with congenital disability worldwide. Having a child with a disability could have profound implications for parents. Available evidence has primarily focused on labor‐market outcomes (Wasi et al. 2012; Gunnsteinsson and Steingrimsdottir 2019; Asuman, Gerdtham, Alriksson‐Schmidt, Nordin, and Jarl 2024a; Breivik and Costa‐Ramón 2024), with little attention paid to other parental outcomes such as mental health. Parenthood may lead to the accumulation of stress that has negative consequences on mental health (Hart et al. 2023). This may be more severe for parents of children with disabilities compared to parents of typically developing children. Having a child with a disability may impose a substantial financial burden on parents due to medical expenses, forcing parents to increase labor‐market participation. Equally, the increased care demands related to having a child with a disability may increase the pressures on parents (Chen et al. 2023; Hart et al. 2023; Cheung et al. 2025). Parents of children with disabilities may, therefore, be at a higher risk of experiencing poor mental health.

Understanding the mental‐health consequences of having a child with a disability is important in its own right, as poor mental health may constitute a welfare loss to parents. Severe mental‐health disorders may increase social alienation, limit social opportunities and participation, and reduce physical health (Becker and Kleinman 2013; Layard 2017). The negative mental health consequences may exert direct costs on families, the healthcare system, and society as a whole through payments for healthcare consultations and dispensed medications. The burden of poor mental health on society may be substantial through the loss of productivity associated with reduced work capacity. Indeed, mental health disorders are the leading cause of reduced productivity in most high‐income countries, accounting for 40% of sickness absence (Shiels et al. 2004; Höglund et al. 2020) and 35% of disability benefits (Organization for Economic Co‐operating and Development 2003). Mental health also provides an avenue to understand the mechanisms behind the different effects of child disability on labor‐market outcomes between parents (Ahammer et al. 2023; Breivik and Costa‐Ramón 2024). Mothers may face biological, social, and institutional factors that increase their risk of mental health disorders after childbirth (Ruppanner et al. 2019). Thus, differences in mental‐health effects of parenting a child with a disability between mothers and fathers may help to explain the observed differences in the labor‐market outcomes between fathers and mothers following the birth of a child with a disability.

In addition, the mental health of parents can also have consequences for the well‐being of the child, who depends on the parents for care and support. Poor parental mental health may lead to negative parenting behaviors that could create an adverse environment for child development (Wilson and Durbin 2010), with potential long‐term consequences (Johnston et al. 2013; Kamis 2021; Bütikofer et al. 2024).

Estimating the causal relationship between a child's disability and the mental health of parents over time is challenging for several reasons. First, mental health is difficult to measure. Most studies have relied on self‐reported measures of mental well‐being, which is prone to respondent biases. This is exacerbated by the limited availability of longitudinal data that captures the mental health of parents prior to the birth of the child. As a result, studies have employed self‐reported outcomes that may capture mental health state after treatment of a mental‐health condition, leading to under‐estimation of the true impacts (Ahammer et al. 2023).

The second challenge arises from finding a suitable *identification strategy*. The timing of childbirth, the likelihood of having a child with a disability, and mental health, may be affected by many factors that are difficult to account for. Particularly, socioeconomic factors may jointly influence mental‐health disorders (Linder et al. 2020) and the likelihood of having a child with a disability (Korzeniewski et al. 2018). Further, differences in mental health may emerge between parents of children with disabilities and typically developing children before childbirth. Thus, the possibility that parental mental health may lead to the birth of a child with a disability complicates accurate estimations of causal effects.

In this paper, we leverage data from Swedish administrative registers and apply an event‐study approach to examine the impact of child disability on the mental health of parents around the timing of first birth following Kleven et al. (2019) and Ahammer et al. (2023). We use data on dispensed medications for mental‐health‐related disorders that enable us to overcome the measurement challenge. Dispensed‐medication data may reflect severe and less severe conditions assessed by professionals (Hart et al. 2023). Most existing studies have focused on single mental‐health disorders, particularly depression or the use of mental healthcare services (Ahammer et al. 2023; Chen et al. 2023; Cheung et al. 2025). We focus on a broad array of mental health disorders and analyze our sample separately for mothers and fathers, thereby deepening our understanding and highlighting nuances in mental health that may exist between parents.

To overcome the identification challenge, we focus on Cerebral Palsy (CP), an early‐onset musculoskeletal disability that affects about 2–3 births per 1000 live births in Europe (Sellier et al. 2016; Hollung et al. 2018; Himmelmann and Uvebrant 2018; Larsen et al. 2021; McIntyre et al. 2022). It is caused by a non‐progressive brain damage to a fetal or infant brain. Previous studies have highlighted unique epidemiological features of CP that facilitate the identification of causal effects (Müller et al. 2022; Asuman, Gerdtham, Alriksson‐Schmidt, Nordin, and Jarl 2024a; Lin et al. 2024). The key advantage of CP arises from the fact that, unlike other congenital and early‐onset disabilities such as spina bifida, Down syndrome, or spinal muscular atrophy (SMA), CP cannot be detected before birth. As such, expecting parents cannot anticipate the birth of a child with CP and selectively choose to terminate a pregnancy. The timing of the birth of a child with CP may, therefore, be plausibly considered exogenous. Further, recent studies such as Müller et al. (2022) and Asuman, Gerdtham, Alriksson‐Schmidt, Nordin, and Jarl (2024a) have also shown that the likelihood of CP is uncorrelated with parental characteristics, including education, income, and mental health in Sweden. Thus, we can rule out reverse causality between mental health and the likelihood of having a child with CP as well as confounding factors such as education and income jointly determining treatment (having a child with CP) and outcome (mental health of parents). Finally, the longitudinal nature of the data enables us to minimize potential biases, as we are able to compare parents before the birth of the child and also account for unobserved time‐invariant characteristics that may affect the outcome under consideration.

With a relatively common prevalence, the focus on CP provides an avenue to examine heterogeneity along several dimensions, such as parental background and disability‐related characteristics. CP is associated with a wide variation of impairments that may have heterogeneous effects on parental mental health, both in terms of type of impairment and severity level. Parents of children with severe impairments are expected to face heightened physical and emotional stressors that may negatively affect their mental health compared to parents of children with mild impairments. Also, access to social support and coping mechanisms may differ across socioeconomic backgrounds. Such differences may result in different mental‐health consequences of having a child with CP across parental background characteristics. To this end, we also examine heterogeneity in the mental‐health consequences of having a child with CP across parental characteristics such as age at birth, education, and income.

Two main findings emerge from this study. First, our results show that dispensed medications for mental‐health‐related conditions increase for parents of children with CP compared to parents of children without CP following the birth of a child. However, differences in the mental‐health conditions for which parents are dispensed medications exist. Particularly, we find that the birth of a child with CP leads to an increase in dispensed medications for psychosis‐ and anxiety‐related disorders for fathers and anti‐depressants for mothers. Second, we find that the mental‐health consequences of having a child with CP do not vary across parental characteristics. However, dispensed medications for mental‐health‐related conditions are higher among parents of children with severe impairments than those with mild impairments.

## 2 The Swedish Healthcare System

Sweden provides universal healthcare with access to high‐quality healthcare for all legal residents. The public healthcare system is financed through regional‐ and municipal‐level taxes and grants from the central government. Mental health care is an integral part of the Swedish public health system and is available to everyone who requires the services. Mental health care is organized and regulated by the same legislation and user benefits as other healthcare services, including user fees (Glenngård 2020). Spending on healthcare visits is centrally regulated, with a maximum spending cap of SEK 1150 (≈ EUR 115) on primary care and outpatient specialist visits per person in a 12‐month period in 2020 (Glenngård 2020). Most common mental‐health conditions are usually treated in the primary healthcare setting by medical practitioners such as doctors, psychologists, or psychotherapists. Severe mental‐health conditions may be referred to specialized psychiatric care in outpatient settings or hospitals. However, even in a context of low‐cost services, healthcare‐seeking behavior is low for mental‐health conditions as only half of the persons who met clinic symptoms of common mental health conditions have been shown to seek care (Wallerblad et al. 2012).

A reimbursement scheme also exists for outpatient prescription drugs and medical devices. Individuals pay the full or part of the amount for covered prescription drugs and medical devices up to an annual maximum of SEK 1150 in 2020 (≈ EUR 115), after which subsidy gradually increase until they reach a spending cap of SEK 2300 (≈ EUR 230) within a 12‐month period in 2020 (Glenngård 2020). Persons aged under 18 years are exempt from co‐payments for prescribed drugs and devices. The cap for prescribed drugs and medical devices is separated from the cap on primary care and outpatient consultations. Individuals pay the full cost for drugs and medical devices not covered by reimbursement schemes, including over‐the‐counter medicines.

## 3 Data

Our data is constructed from several Swedish administrative registers. First, we identified all persons living with CP in Sweden between 1990 and 2015 using ICD code G80 from the National Patients Register (NPR), the National Quality Register and Follow‐up Program for Individuals with Cerebral Palsy (CPUP), and the Medical Births Register (MBR).^1^ We then generate a 5:1 control group of persons without CP from the Register of the Total Population match on sex, year of birth, and municipality of residence.^2^ Parents of both groups of children are then identified from the Multi‐generational Register. Our control group is chosen from the general population and may include children with other disabilities.^3^ Children with CP may have other disabilities that are not the result of the underlying brain damage that causes CP (Reid et al. 2018; Påhlman et al. 2019). As such, comparing persons with CP to a *clean* control group risk over‐estimating the impacts of having a child with CP on parents. A “general population” control group allows us to estimate the effect in comparison to the average population—that is, what could be expected to be the average outcomes in absence of CP.

We linked parents to other registers using personal identification numbers. We obtain data on dispensed medications from the Prescribed Drugs Register, which contains information on all prescribed drugs dispensed in pharmacies in Sweden. The register started in July 2005; thus, we have information for a full year starting January 2006. Information on socio‐demographic characteristics and disposable income are obtained from Statistics Sweden's Longitudinal Integrated Database for Health Insurance and Labor Market Studies (*Swedish: Longitidinell Intergrationsdatabas för Sjukförsäkrings och Arbetsmarknadsstudier*, LISA), a longitudinal database comprising detailed data for all individuals older than 15 years. Information on secondary conditions, comorbidities, and CP‐specific characteristics is obtained from the National Patients‐ (NPR) and CPUP Registers. Finally, data on healthcare utilization is obtained from the NPR, containing information on all completed inpatient stays, specialized outpatient care, and admissions to compulsory psychiatric care, but does not include information on primary care and treatment by professionals other than doctors.

We then restrict our sample to parents of children born in Sweden between 2009 and 2015, which allows us to construct a balanced panel of parents from 2006 to 2020. This means we can follow the same parents from three years before the child's birth to five years after. We focus on first births to overcome potential biases due to endogeneity of subsequent births. We identify the birth order of the child from the Medical Births Register for mothers. However, we cannot identify whether the child is the first‐born of the father. This is because mothers and babies are register at the time of birth, and as a result we identify more mothers than fathers in our sample. Our final sample consists of 3988 matched father‐child pairs, of which 573 (14.4%) have CP, and 4091 matched mother‐child pairs, of which 594 (14.5%) have CP.

We measure mental‐health outcomes with dispensed medication for specific mental‐health conditions, focusing on psychotropic medications. These medications are generally used for the treatment of disorders such as schizophrenia, anxiety, insomnia, and depression. The conditions for which these medications are dispensed are among the most common mental‐health disorders (Santomauro et al. 2021) with prevalence estimated to be one‐third of the working‐age population in Europe (Wittchen et al. 2011). In Sweden, these conditions have been prioritized as a policy area (Jarl et al. 2020) as they constitute the most common cause of sick leave (Höglund et al. 2020).

Dispensed medications for mental‐health disorders offer a comprehensive measure of mental health as they may occur anywhere in the healthcare system: primary care, outpatient, or inpatient care (Linder et al. 2023). In addition, dispensed medications also capture treatment for ongoing diagnoses. Using Anatomical Therapeutic Chemical (ATC) Classification level‐4 codes, we identify four classes of medications from the Prescribed Drugs Registers: *anti‐psychotics* (ATC code N05A), *anxiolytics* (ATC code N05B), *hypnotics and sedatives* (ATC N05C), and *antidepressants* (ATC code N06A). For our analysis, we begin by generating a composite indicator of mental health by combining these four types of drugs. This variable takes the value of 1 if an individual was dispensed any of the medications in a year and 0 otherwise. We then proceed to examine each class of medication separately. In this paper, we estimate the extensive margin of the mental‐health effects of having a child with CP.

Table 1 shows the parental characteristics 2 years before the child's birth. Fathers of children with and without CP appear to be statistically comparable in many characteristics ‐ healthcare visits, dispensed medications for mental health‐related conditions, educational attainment, and labor‐market outcomes. Although mothers of children with and without CP are statistically similar on many indicators, mothers of children with CP are dispensed fewer medications for anti‐depressants and are less likely to be employed 2 years before the birth of the child.

**TABLE 1:** Parental characteristics 2 years before birth.

	Fathers			Mothers		
Variables	With CP	Without CP	Difference	With CP	Without CP	Difference
Parental year of birth	1979.63	1979.58	0.047	1982.54	1982.58	0.04
Child's year of birth	2011.68	2011.49	0.18**	2011.68	2011.50	0.18 **
Inpatient care (%)	0.01	0.01	0.00	0.003	0.011	0.008
Outpatient care (%)	0.09	0.07	0.02	0.116	0.119	0.003
Any mental health (%)	6.63	5.51	1.12	9.43	9.58	0.15
Anti‐psychotics (%)	0.00	0.44	0.44	0.67	0.63	0.04
Anxiolytics (%)	1.57	1.90	0.33	4.21	3.17	1.03
Hypnotics & sedatives (%)	2.09	2.69	0.60	3.54	3.15	0.39
Anti‐depressants (%)	4.36	3.16	1.20	4.55	6.98	2.43**
Married/partner (%)	21.61	21.10	0.50	21.31	20.04	1.27
Mandatory education (%)	14.37	12.86	1.50	13.53	10.99	2.54*
Secondary education (%)	48.39	47.59	0.81	36.01	37.73	1.64
Higher education (%)	37.24	39.55	2.31	50.38	51.27	0.89
Employed (%)	89.56	91.68	2.12	86.70	89.95	3.25**
Earnings (1000SEK)	300.360	295.174	5.185	220.788	226.371	5.583
Disposable income (1000SEK)	222.918	229.500	6.582	185.012	177.661	7.351

*Note:* All characteristics are measured 2 years prior to birth of the child to avoid the characteristics being impacted by childbirth and CP status of the child. All monetary values are reported in 1000 SEK adjusted to 2015 level using the CPI. ***p < 0.01, **p < 0.05, *p < 0.1.

To overcome the risk of Type‐1 error when testing multiple outcomes, we conduct a multiple hypothesis test based on the Romano and Wolf (2016) correction of test differences in parental characteristics, including mental health 2 years prior to the birth of the child. We do not find any systematic differences between parents prior to the birth of a child with CP (see Table 2), which boosts our suggestion that having a child with CP may be exogenous to parental characteristics.

**TABLE 2:** Adjusted *p*‐*values* for multiple hypothesis testing.

	Fathers		Mothers	
Outcome	Model *p*‐value	RW *p*‐value	Model *p*‐value	RW *p*‐value
Parental year of birth	0.872	0.997	0.871	1.000
Child's year of birth	0.033	0.529	0.031	0.421
Inpatient care	0.718	0.997	0.311	0.976
Outpatient care	0.442	0.993	0.945	1.000
Any mental health	0.281	0.977	0.907	1.000
Anti‐psychotics	0.112	0.807	0.900	1.000
Anxiolytics	0.585	0.997	0.194	0.894
Hypnotics & sedatives	0.405	0.993	0.618	0.995
Anti‐depressants	0.139	0.859	0.028	0.398
Married/partner	0.790	0.997	0.493	0.989
Mandatory education	0.342	0.992	0.087	0.667
Secondary education	0.731	0.997	0.469	0.989
Higher education	0.313	0.984	0.701	0.998
Employed	0.102	0.807	0.022	0.359
Earnings	0.857	0.997	0.073	0.635
Disposable income	0.421	0.993	0.304	0.976

*Note:* This table shows the *p*‐values associated with the estimated differences between parents of children with CP and the control 2 years before the birth of the child. The RW *p*‐value adjusts for multiple hypothesis testing using the Romano and Wolf (2016) correction with 1000 replications.

## 4 Methodology

### 4.1 Estimation Strategy and Identification

We conduct an event study of the form;

(1) $$y_{\mathrm{ist}}=\sum_{\begin{array}{c} j=-3\\ j\neq -2 \end{array}}^{+5}\beta_{j}\cdot I_{it}\left(j=t\right)+\sum_{\begin{array}{c} j=-3\\ j\neq -2 \end{array}}^{+5}\delta_{j}\cdot I_{it}\left(j=t\right)\cdot I_{i}\left(CP=1\right)+\sum_{k}\phi_{k}\cdot I_{it}\left(k=age_{is}\right)+\sum_{y}\omega_{y}\cdot I_{it}\left(y=s\right)+\mu_{i}+\epsilon_{\mathrm{ist}}$$

where $y_{\mathrm{ist}}$ is the mental‐health outcome of interest of parent i at calendar year s and event time t. The first term on the right‐hand side of Equation (1) captures event‐time dummies, where we define event time as the years relative to the birth of the child, so t=0 is the year of birth. To avoid endogeneity, the reference period is set before the birth of the child. However, due to the potential risks of taking certain medications before and during pregnancy, persons may be taken off medications in the year preceding or during pregnancy. We, therefore, use the event time t=−2 as our reference period.

The second term contains interaction dummies between event time and having a child with CP, where CP=1 if parents have a child with CP and CP=0 otherwise. In Equation (1), the β coefficients measure the effect of having a child on the mental health of parents, while the δ coefficients capture the difference in mental‐health outcomes between parents having a child with or without CP. The total effect of having a child with CP is measured by β+δ. We focus on the difference in dispensed mental health medications (δ) between parents in our presentation of main results^4^. The third term is age dummies, which account for life‐cycle effects in mental health, while the fourth term reflects year dummies, which control for time trends in dispensed medications. $\mu_{i}$ captures individual fixed effects and $\epsilon_{\mathrm{ist}}$ is a random error term. Standard errors are robust and clustered at the individual level. We estimate Equation (1) separately for mothers and fathers, as the effects of pregnancy, childbirth, and parenting differ substantially between women and men. Particularly, postpartum depression, a common complication of childbirth, has been found to lead to an increase in depression and anxiety among mothers in the first year after birth (Stewart and Vigod 2019).

A causal interpretation of our estimates will require that we satisfy three identification assumptions. First is the *parallel trends* assumption, requiring that the mental health of parents with and without a disabled child would have evolved similarly after the birth of the child in the absence of having a child with CP. The second assumption requires that having a child with CP is uncorrelated with parental characteristics, which has been shown in previous studies using similar samples from Swedish administrative registers, Müller et al. (2022) and Asuman, Gerdtham, Alriksson‐Schmidt, Nordin, and Jarl (2024a). The results of our multiple hypothesis test (Table 2) confirm that parents are not systematical different prior to the birth of the child with CP. Further, CP is also associated with relatively severe functional impairments that are very unlikely to be undiagnosed in the Swedish healthcare system. Thus, it is safe to assume that CP is plausibly random in Sweden, and diagnosis is uncorrelated to parental socioeconomic status. The last assumption requires that there is no anticipatory behavior among parents regarding having a child with CP. Although postnatal events may result in brain damage that leads to CP, the underlying brain damage of CP occurs mostly at birth and is undetectable *in utero*. As such, parents are unlikely to select to have a child CP.

### 4.2 Heterogeneity Analyses

Substantial variations in severity and function exist among persons with CP. While some persons may function independently, others may depend entirely on help to undertake basic activities of daily life (Andrén and Grimby 2004). As such, the mental health consequences of having a child with CP may differ across the severity of impairments. In addition, parental mental responses may differ across parental characteristics. Parents with higher socioeconomic status may have better resources and opportunities to cope with childcare demands and associated stressors and strains and seek treatment (Ryu and Fan 2023). We, therefore, proceed to examine whether the mental‐health effects of having a child with CP vary across parental characteristics and the severity of impairments. For mothers and fathers separately, we re‐estimate Equation (1) based on socioeconomic characteristics and severity of impairments.

We adopt three proxies for parental socioeconomic status: age at (first) birth, educational attainment, and individual‐level disposable income. We group parents into young or old based on our sample's median age at birth.^5^ We measure educational attainment and disposable income 2 years prior to the birth of the child. Parents are grouped into lower educated if their highest educational attainment is mandatory or secondary education and higher educated if they have completed higher education. Finally, parents with disposable income below the median are classified *low income* and those with disposable income greater than the median disposable as *high income*.

Further, co‐residence with a partner may provide avenue to share the burdens of parenting for a child with CP and serve as a social support mechanism. Co‐residence with a partner has been shown to reduce the risks of poor health‐mental outcomes (Giannelis et al. 2021; Zhai et al. 2024; Gueltzow et al. 2024). Our data includes information on co‐residence with a partner in the year of birth of the child. We therefore proceed to examine whether the mental‐health consequences of having a child with CP differs across co‐residency status of parents. To do this, we classify parents to be living alone or co‐residing with a partner based on the family situation as registered in the LISA database.

Next, we examine whether parental mental‐health consequences differ by the severity of CP. The two most common classifications of severity of CP are the Gross Motor Function Classification System (GMFCS) and the Manual Ability Classification System (MACS). Both classify the severity of CP into five levels where higher levels correspond to greater functional impairment (Compagnone et al. 2014). While GMFCS is based on self‐initiated movement abilities (Palisano et al. 2008), MACS classifies severity based on the ability to use hands to handle daily‐life activities (Eliasson et al. 2006). We classify the severity of CP based GMFCS and MACS levels closest to the age of five. Children with GMFCS and MACS levels I and II are classified as mild, and levels III to V are classified as severe. GMFCS I and II correspond to ability to self‐initiate movements, while GMFCS III to V require assistive device (Compagnone et al. 2014). Children with MACS levels I and II are able to handle objects easily or with reduced quality, while others with levels III to V handle objects with difficulty or severe impairment to perform everyday tasks. Using both classification systems provides us with an avenue to validate our results. Of the children with CP in our data, 65%, and 62% are classified as mild based on GMFCS and MACS levels, respectively.

### 4.3 Other Outcomes: Robustness and Mechanisms

It is important to understand whether the differences in dispensed medications for mental health‐related conditions between parents of children with and without CP reflect actual differences in mental health due to parenting a child with CP. Particularly, differences in dispensed medications for mental health‐related conditions may reflect differences in health‐seeking behaviors as parents of children with CP are in much closer contact with the healthcare system due to the medical and other healthcare needs of the child. Though such healthcare visits focus on the child, they include assessments of the home situation and the parents' ability to care for the child. Health professionals may notice the mental state of parents during these child‐health visits and can recommend or advise parents to seek diagnoses and treatment. To explore this possibility, we compare the likelihood and frequency of healthcare visits in inpatient and outpatient care between parents of children with and without CP.

It is also possible that having a child with CP may affect parents' health behaviors. In one way, parents may adopt addictive behaviors to cope with the stress and demands of caring for a child with CP. On the other hand, the increased responsibility of caring for a child with CP may cause some parents who were previously using addictive substances to seek treatment. To investigate this hypothesis, we adopt two approaches. First, we test whether parents receive diagnoses for a mental or behavioral disorder due to psychoactive substance abuse (ICD‐10 code: F10‐19) in either out‐ or in‐patient care. Second, we examine whether dispensed medications for treating addiction related to nicotine, alcohol, and opioids (*ATC code: N07B*) change after the birth of a child with CP.

Lastly, we examine the cost implications of dispensed medications for mental health conditions after the birth of a child with CP. Mental‐health services and medications are covered under the publicly funded healthcare system. However, individuals pay the full costs of dispensed medications until they reach an annual maximum amount, after which subsidies gradually increase to 100%. We measure the cost of medications as the annual patient costs, excluding taxes.

## 5 Results

### 5.1 Dispensed Medications for Any Mental‐Health Disorders

**FIGURE 1:**
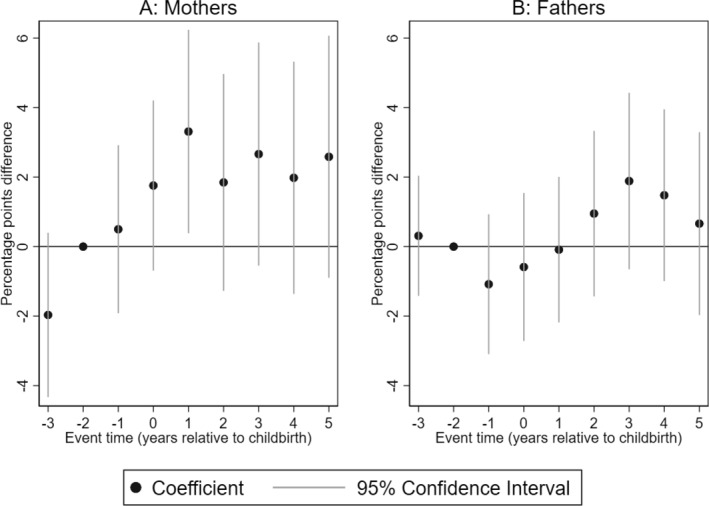
Impact of having a child with CP on mental health‐related medications. Event study estimates for mothers (Panel A) and fathers (Panel B). The outcome is dispensed medication related to anxiety, sleep disorders, and depression. The plot shows the coefficients of the interaction terms between event times and child CP status estimated by Equation (1). Spikes show the 95% confidence intervals. The regression includes controls for event‐time dummies, calendar year, and age dummies.

We begin by presenting the impact of having a child with CP on the likelihood of a parent being dispensed medication for a mental‐health‐related condition. Figure 1 shows that 1 year after birth, mothers of children with CP are 3.3% points more likely to be dispensed medication related to anxiety, sleep disorders, and depression compared to mothers of children without CP (Panel A). Scaled by the mean of mothers of children without CP in the reference year, this is equivalent to a 36% increase. Although this effect is only statistically significant in the first year after birth, the trend suggests a persistent increase in dispensed medications for mental‐health conditions in subsequent years. For fathers, we do not find statistically significant differences in the likelihood of being dispensed medication for a mental health condition in the period after the birth of the child.

### 5.2 Dispensed Medications for Specific Mental‐Health Disorders

Our composite indicator of mental health shown in Figure 1 may mask the impacts of having a child with CP on specific mental‐health conditions. We, therefore, proceed to examine the impact of having a child with CP on specific mental health conditions.

#### 5.2.1 Anti‐Psychotics

**FIGURE 2:**
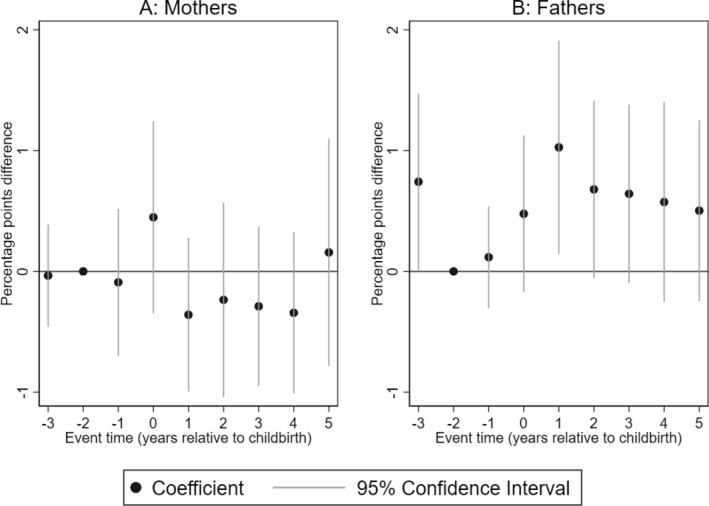
Impact of having a child with CP on dispensed anti‐psychotics. Event study estimates for mothers (Panel A) and fathers (Panel B). The outcome is dispensed anti‐psychotic. The plot shows the coefficients of the interaction terms between event times and child's CP status estimated by Equation (1). Spikes show the 95% confidence intervals. The regression includes controls for event‐time dummies, calendar year, and age dummies.

We begin by looking at dispensed anti‐psychotics used for the treatment of mental‐health conditions that include psychotic experiences. Our results show that dispensed anti‐psychotics medications are not statistically different between mothers of children with and without CP (see Figure 2). However, the point estimate for mothers of children with CP is slightly raised in the year of birth of the child. For fathers, the likelihood of being dispensed anti‐psychotics medications increases in the period after the birth of the child with CP. Particularly, the likelihood increases by one percentage point, equivalent to a 227% increase over the mean dispensed medications for fathers of children without CP in the reference year. The point estimate remains elevated, albeit not statistically significantly different from zero in the following 4 years.

#### 5.2.2 Anxiolytics

**FIGURE 3:**
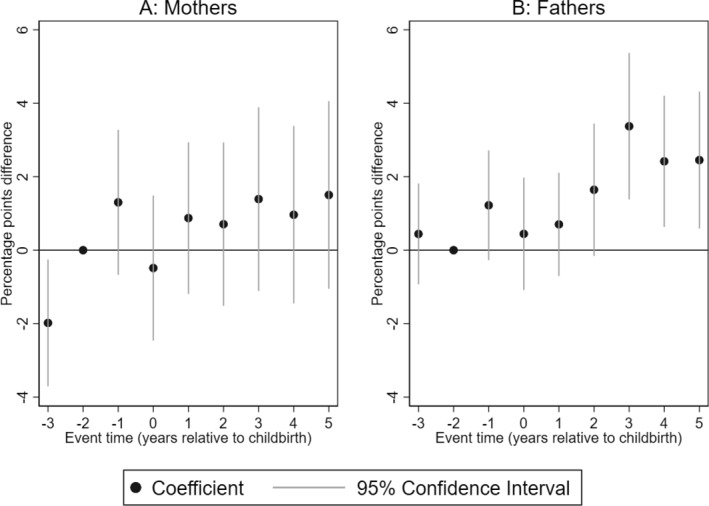
Impact of having a child with CP on dispensed anxiolytics. Event study estimates for mothers (Panel A) and fathers (Panel B). The outcome is dispensed anxiolytics for anxiety‐related conditions. The plot shows the coefficients of the interaction terms between event times and child's CP status estimated by Equation (1). Spikes show the 95% confidence intervals. The regression includes controls for event‐time dummies, calendar year, and age dummies.

In Figure 3, we show the impact of having a child with CP on dispensed anxiolytics for the treatment of anxiety‐related conditions. We find that although mothers of children with CP are less likely to be dispensed anxiolytics 3 years prior to the birth of the child, we do not find statistically significant impacts in the period after the birth of the child (Panel A). However, we find that the likelihood of fathers of children with CP being dispensed anxiolytics increases by 3.4% points in the third year after the birth of the child and stabilizes at 2.4% points in the fourth and fifth years after birth. Relative to the mean of fathers of children without CP in the reference year, this is equivalent to a 126%–179% increase in the likelihood of fathers being dispensed anxiety‐related medications.

#### 5.2.3 Hypnotics and Sedatives

**FIGURE 4:**
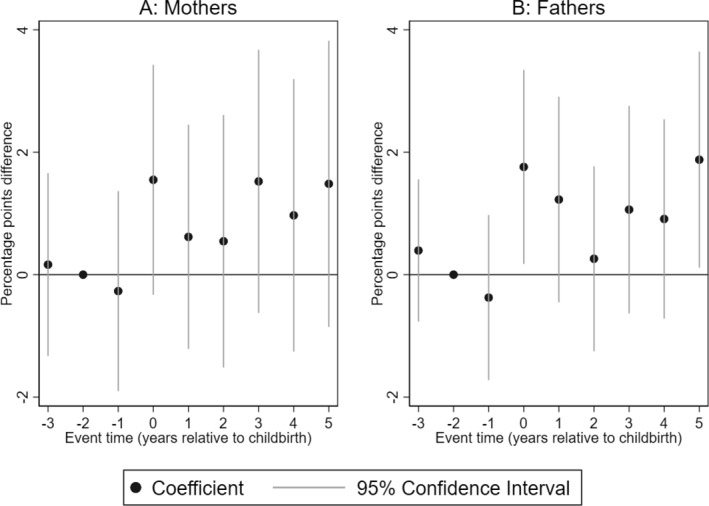
Impact of having a child with CP on dispensed hypnotics and sedatives. Event study estimates for mothers (Panel A) and fathers (Panel B). The outcome is dispensed hypnotics and sedatives used for the treatment of sleep disorders. The plot shows the coefficients of the interaction terms between event times and child's CP status estimated by Equation (1). Spikes show the 95% confidence intervals. The regression includes controls for event‐time dummies, calendar year, and age dummies.

We examine next the impact of having a child with CP on dispensed hypnotics and sedatives used for parents. We find that although mothers of children with CP are likely to be dispensed hypnotics and sedatives in the years after the birth of the child, these effects are largely statistically insignificant (see Figure 4). For fathers, however, we find statistically significant increases in dispensed hypnotics and sedatives in the year of birth of the child and 5 years afterward, with elevated albeit not statistically significant point estimates in the years in between.

#### 5.2.4 Anti‐Depressants

**FIGURE 5:**
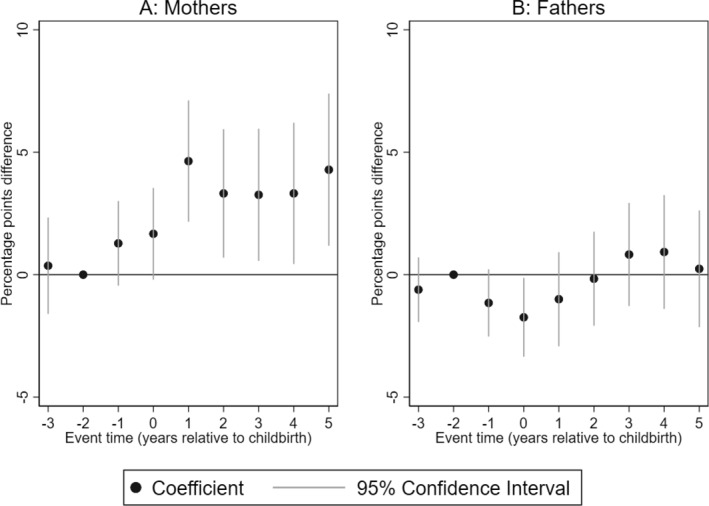
Impact of having a child with CP on dispensed anti‐depressants. Event study estimates for mothers (Panel A) and fathers (Panel B). The outcome is dispensed anti‐depressants. The plot shows the coefficients of the interaction terms between event times and child's CP status estimated by Equation (1). Spikes show the 95% confidence intervals. The regression includes controls for event‐time dummies, calendar year, and age dummies.

Next, we examine the impact of having a child with CP on dispensed anti‐depressants. Figure 5 shows the impact of having a child with CP on dispensed anti‐depressants. In the first year after birth, the likelihood of mothers of children with CP being dispensed anti‐depressants increases by 4.6% points. It stabilizes at 3.3% points between the second and fourth years after the child's birth. This is a 66.5% increase in the first year and 47% between the second and fourth years after the birth of the child over the mean dispensed anti‐depressants for mothers of children without CP in the reference year. By the fifth year after the birth of the child, mothers of children with CP are 4.4% points (64% over the reference year for mothers of children without CP) more likely to be dispensed anti‐depressants than mothers of children without CP. On the other hand, we find fathers of children with CP to be 1.7% points less likely to be dispensed anti‐depressants in the year of the birth of the child. This is equivalent to a 54% decrease over the mean dispensed anti‐depressants of fathers of children without CP.

### 5.3 Heterogeneity Analyses

**FIGURE A2:**
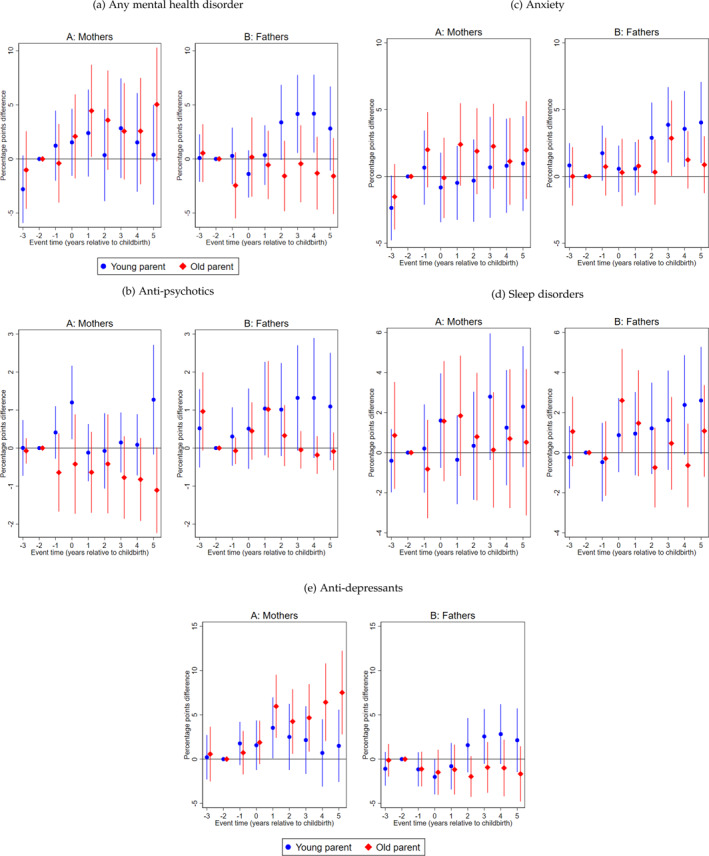
Impact of having a child with CP on parental mental health. Event study estimates for mothers (Panel A) and fathers (Panel B). The outcomes are dispensed medications related to various mental‐health disorders. The plot shows the coefficients of the interaction terms between event times and child CP status estimated by Equation (1) and disaggregated by parental age at birth. Spikes show the 95% confidence intervals. The regression includes controls for event‐time dummies, calendar year, and age dummies.

**FIGURE A3:**
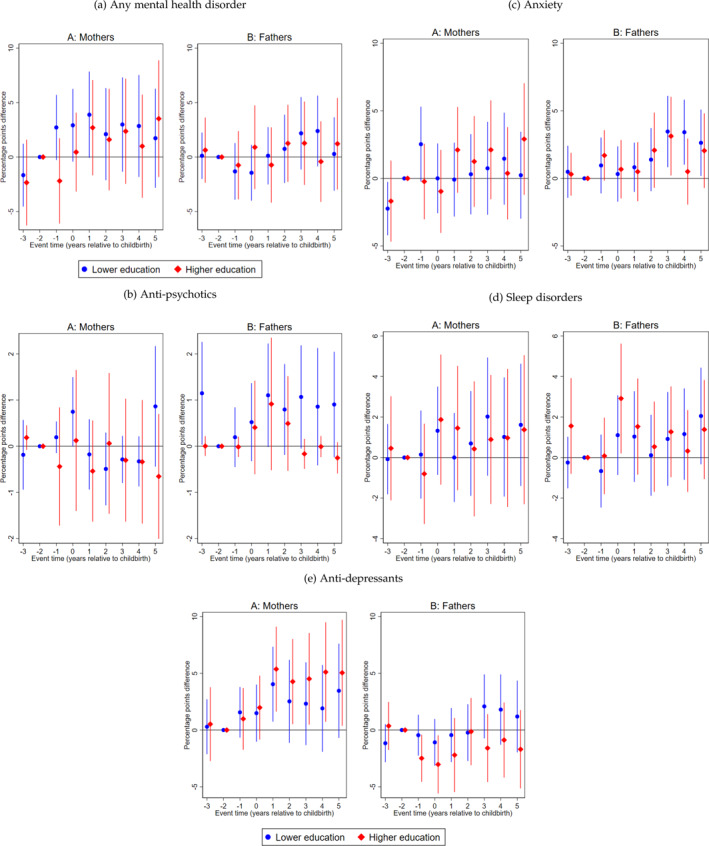
Impact of having a child with CP on parental mental health. Event study estimates for mothers (Panel A) and fathers (Panel B). The outcomes are dispensed medication related to various mental‐health disorders. The plot shows the coefficients of the interaction terms between event times and child CP status estimated by Equation (1) and disaggregated by parental educational attainment 2 years prior to the birth of the child. Spikes show the 95% confidence intervals. The regression includes controls for event‐time dummies, calendar year, and age dummies.

**FIGURE A4:**
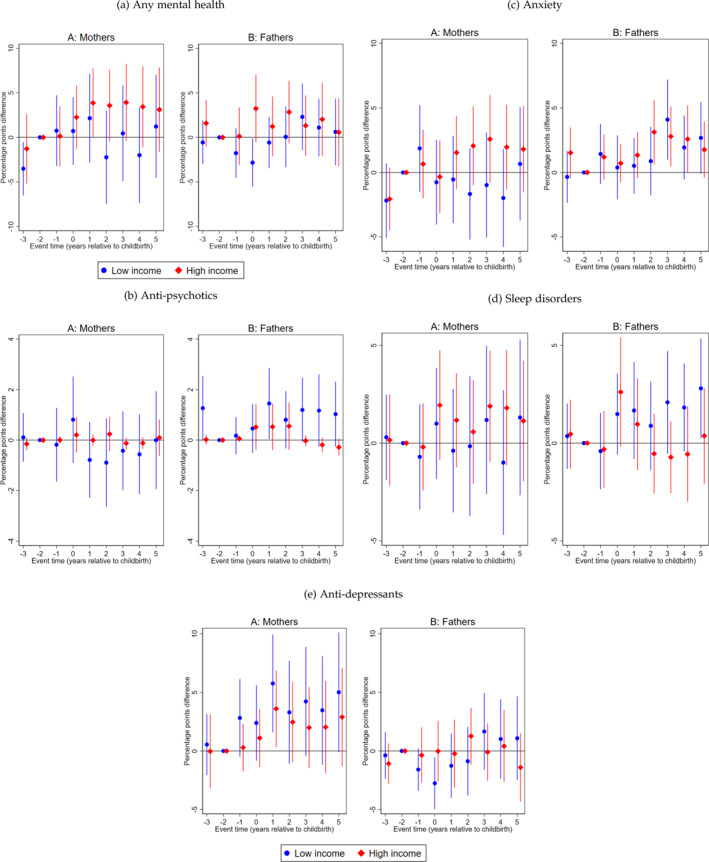
Impact of having a child with CP on parental mental health. Event study estimates for mothers (Panel A) and fathers (Panel B). The outcomes are dispensed medications related to various mental‐health disorders. The plot shows the coefficients of the interaction terms between event times and child CP status estimated by Equation (1) and disaggregated by parental disposable 2 years prior to the birth of the child. Spikes show the 95% confidence intervals. The regression includes controls for event‐time dummies, calendar year, and age dummies.

**FIGURE A5:**
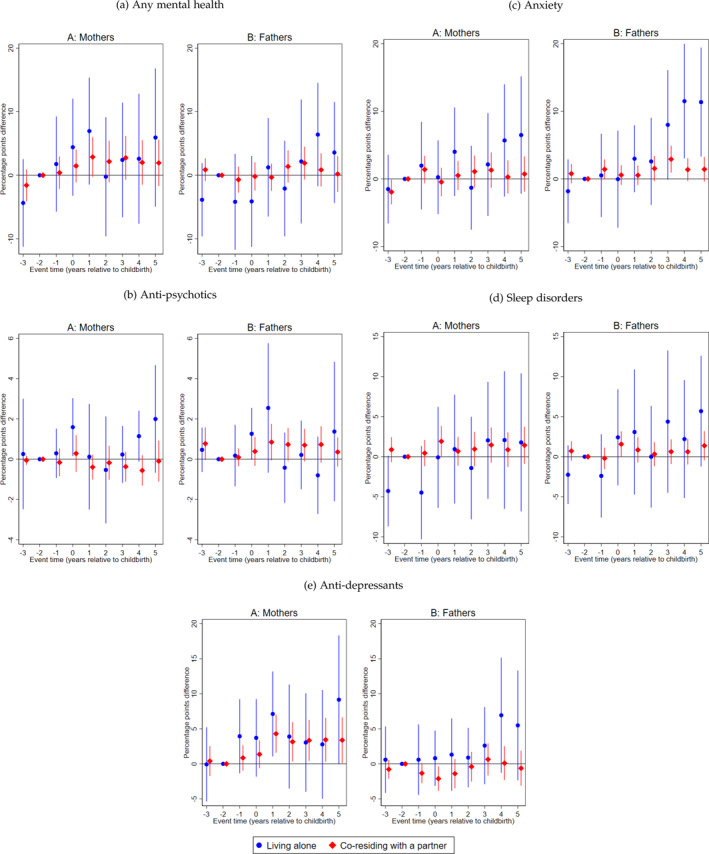
Impact of having a child with CP on parental mental health. Event study estimates for mothers (Panel A) and fathers (Panel B). The outcomes are dispensed medications related to various mental‐health disorders. The plot shows the coefficients of the interaction terms between event times and child CP status estimated by Equation (1) and disaggregated by parental disposable 2 years prior to the birth of the child. Spikes show the 95% confidence intervals. The regression includes controls for event‐time dummies, calendar year, and age dummies.

The results presented thus far might hide heterogeneous effects of having a child with CP across parental characteristics and severity of impairments. We begin by examining heterogeneity across parental socioeconomic characteristics: age at birth, education, and disposable income. The results are attached as Appendix B to this paper (see Figure A2, A3, A4). Overall, the mental‐health effects of having a child with CP appear to be a general phenomenon that only differ marginally across parental characteristics. For example, older and higher‐educated mothers drive the increase in dispensed anti‐depressants for mothers, while younger and lower‐educated fathers drive the increase in dispensed anxiolytics for anxiety‐related conditions. Further, our results suggest that mothers and fathers who living alone are likely to be prescribed anxiolytics compared to those co‐residing with a partner (Figure A5).

**FIGURE A6:**
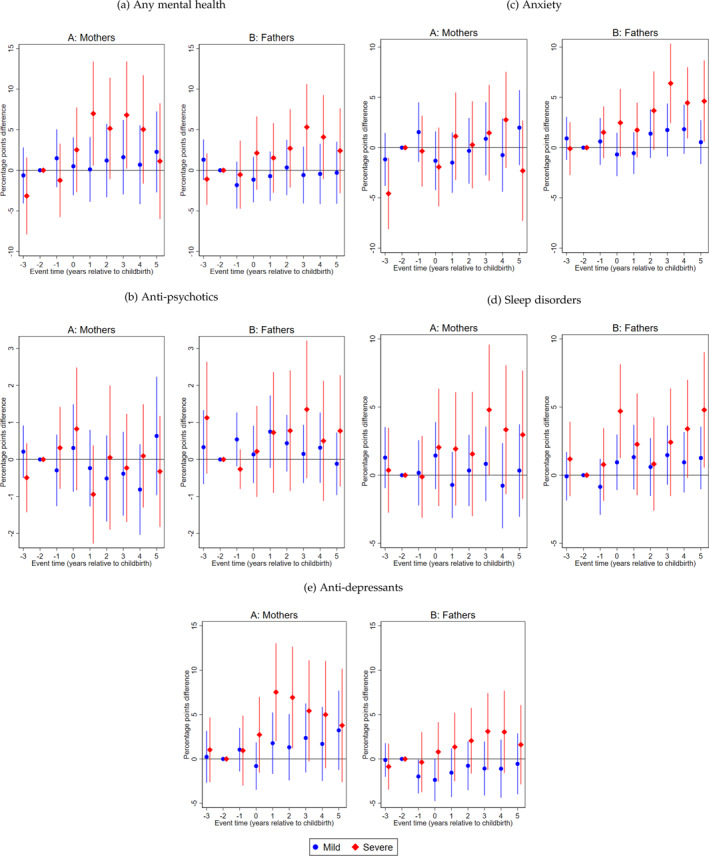
Impact of having a child with CP on parental mental health. Event study estimates for mothers (Panel A) and fathers (Panel B). The outcomes are dispensed medications related to various mental‐health disorders. The plot shows the coefficients of the interaction terms between event times and child CP status estimated by Equation (1) and disaggregated by gross motor functioning ability of the child with CP. Spikes show the 95% confidence intervals. The regression includes controls for event‐time dummies, calendar year, and age dummies.

**FIGURE A7:**
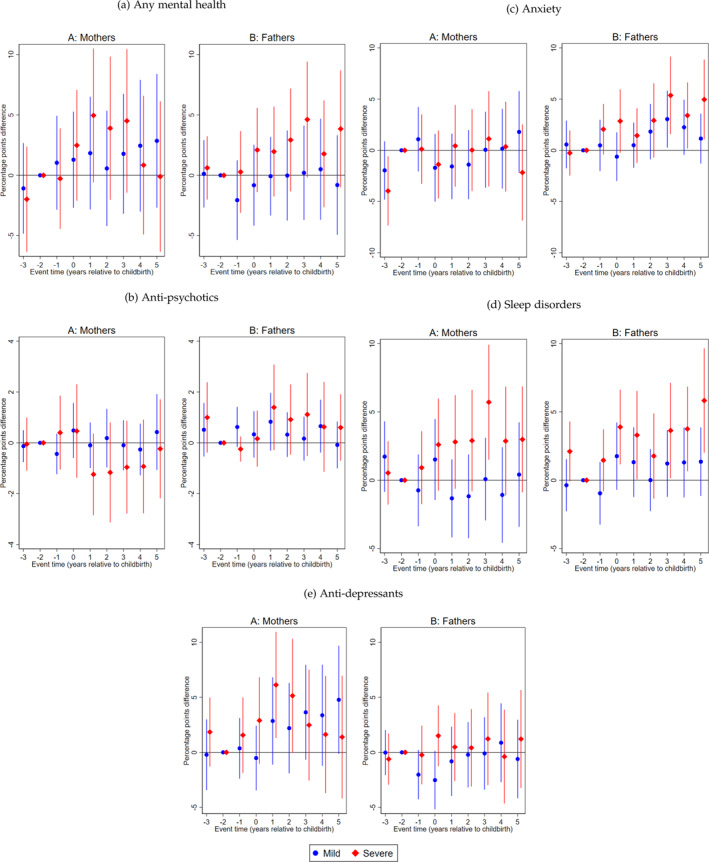
Impact of having a child with CP on parental mental health. Event study estimates for mothers (Panel A) and fathers (Panel B). The outcomes are dispensed medications related to various mental‐health disorders. The plot shows the coefficients of the interaction terms between event times and child CP status estimated by Equation (1) and disaggregated by manual ability of the child with CP. Spikes show the 95% confidence intervals. The regression includes controls for event‐time dummies, calendar year, and age dummies.

Next, we examine heterogeneity across severity of impairments. The results are attached as Appendix B (see Figures A6 and A7). As expected, we find that dispensed medications for mental‐health‐related conditions are generally higher among parents of children with severe impairments than those with mild impairments. The results are robust to the measure of severity adopted, which may not be surprising as GMFCS and MACS are highly correlated (Compagnone et al. 2014). Our results may indicate potential heterogeneous effects across parental characteristics and severity of impairments, although lack of *statistical power* should be kept in mind.

### 5.4 Other Outcomes: Robustness and Mechanisms

#### 5.4.1 Health‐Seeking Behaviors and Healthcare Utilization

**FIGURE A8:**
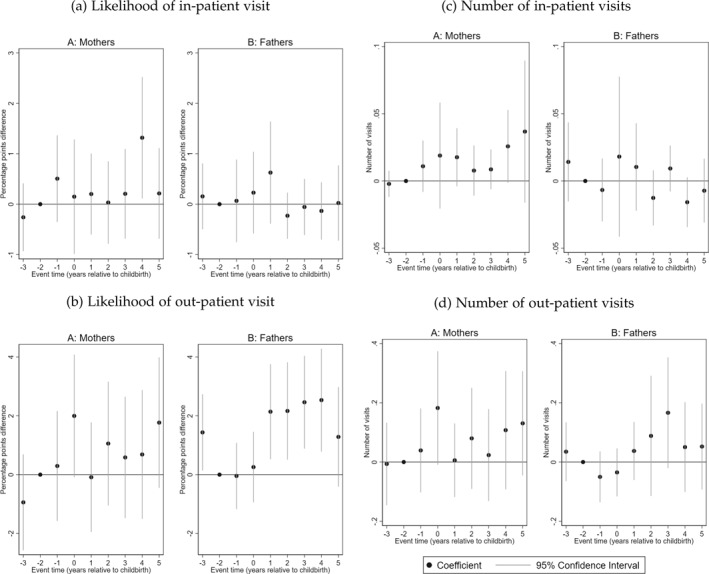
Parental healthcare utilization. Event study estimates for mothers (Panel A) and fathers (Panel B). The outcomes are the likelihood and frequency of use of in‐ and out‐patient healthcare visits. The plot shows the coefficients of the interaction terms between event times and child CP status estimated by Equation (1). Spikes show the 95% confidence intervals. The regression includes controls for event‐time dummies, calendar year, and age dummies.

We examined the likelihood and frequency of in‐ and out‐patient care visits to understand whether the differences in dispensed medications reported in the preceding subsection reflect differences in health‐seeking behaviors and healthcare use. Our results, shown in Appendix C: Figure A8, indicate that fathers of children with CP have an increased likelihood of outpatient care, while no statistically significant effects are noted for frequency of visits or inpatient care, and no notable effects are found for mothers. The results suggest that the increased contact with the healthcare system does not increase parental healthcare visits.

#### 5.4.2 Substance Abuse and Addiction

**FIGURE A9:**
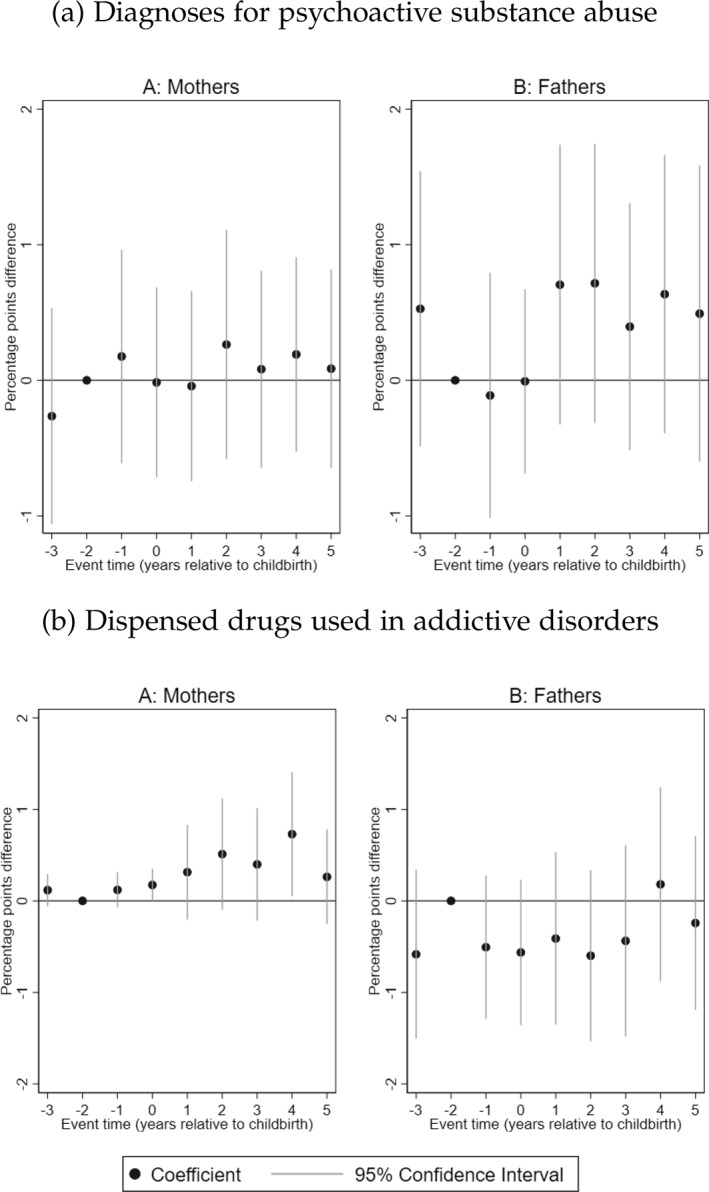
Impact of having a child with CP on parental addiction behavior. Event study estimates for mothers (Panel A) and fathers (Panel B). The outcomes are diagnosis for psychoactive substance abuse and dispensed anti‐addiction medications. The plot shows the coefficients of the interaction terms between event times and child CP status estimated by Equation (1). Spikes show the 95% confidence intervals. The regression includes controls for event‐time dummies, calendar year, and age dummies.

Figures A9a and A9b in Appendix C trace the trajectories of diagnoses of psychoactive substance abuse and dispensed medications for the treatment of addiction disorders, respectively. We find that while fathers of children with CP are more likely to be diagnosed with psychoactive substance abuse, they are less likely to be dispensed anti‐addiction medications. The opposite was noted for mothers. As diagnoses should be recorded at the first healthcare visit, subsequent prescriptions, and medications may be dispensed without a new diagnosis being recorded. However, these effects are not statistically significant. Thus, it is likely that diagnoses will capture new cases while dispensed medications are better at capturing ongoing treatments. It is important to note that these results are not driven by addiction to nicotine or drugs for nicotine dependence. Taken together, the results suggest that having a child with CP may affect parental substance abuse and addiction in opposite ways. While fathers may resort to the use of psychoactive substances to cope with the stresses of parenting a child with CP, mothers, on the other hand, may seek treatment to curb previous addiction due to the responsibilities of caring for a child with CP.

#### 5.4.3 Cost of Dispensed Medications

**FIGURE A10:**
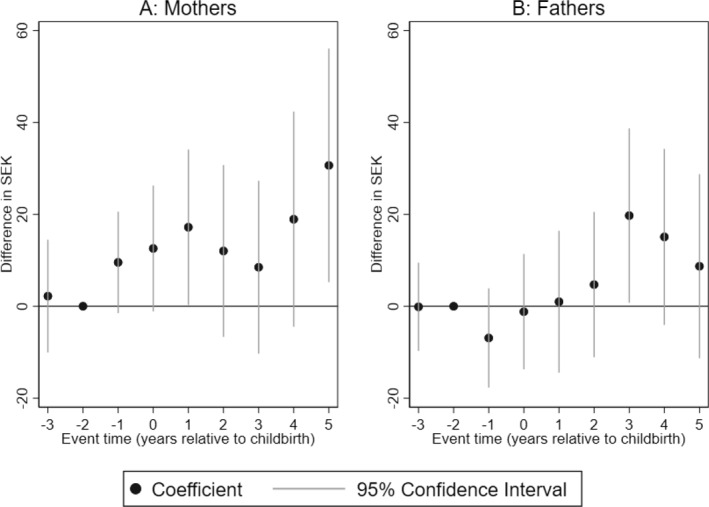
Impact of having a child with CP on dispensed medication cost. Event study estimates for mothers (Panel A) and fathers (Panel B). The outcome is the total cost of dispensed medications for mental‐health‐related conditions. The plot shows the coefficients of the interaction terms between event times and child CP status estimated by Equation (1). Spikes show the 95% confidence intervals. The regression includes controls for event‐time dummies, calendar year, and age dummies.

Lastly, we present the results of the cost of dispensed medications for mental‐health conditions due to having a child with CP. Figure A10 shows an upward trajectory of the medication costs. Mothers of children with CP spend SEK 30 (≈ EUR 3) more in medication costs compared to mothers of children without CP by the fifth year after birth, while fathers incur SEK 20 (≈ EUR 2) in the third year after the birth of the child. This difference in cost is economically insignificant.

## 6 Discussions

We use a long panel of high‐quality data from Swedish administrative registers to investigate the effects of having a child with CP on the mental health of parents. CP is an early‐onset motor disability with unique epidemiological features, and apply an event study to estimate the causal effects of child disability on parental mental health. Our approach compares the trajectories of dispensed medications for mental‐health‐related conditions between parents of children with CP to a control group of parents of children without CP.

Asuman, Gerdtham, Alriksson‐Schmidt, Nordin, and Jarl (2024a) showed that having a child with CP affects parental labor‐market outcomes. The current study shows that the consequences also extend to parental mental health. Dispensed medications for mental‐health disorders increase for parents of children with CP after the birth of the child. However, nuances exist in dispensed medications for mothers and fathers. Compared to parents of children without CP, fathers of children with CP are significantly more likely to be dispensed anti‐anxiety medications. In contrast, mothers of children with CP are significantly more likely to have dispensed anti‐depressants. Thus, while the findings of Asuman, Gerdtham, Alriksson‐Schmidt, Nordin, and Jarl (2024a) may suggest increases in the labor‐market outcomes of parents after the birth of a child with CP, this might be at the expense of their mental health.

Our results further suggest the mental‐health consequences of having a child are a general phenomenon that does not differ significantly by parental characteristics. However, we find that parents of children with severe impairments generally are more likely to be dispensed mental‐health‐related medications than parents of children with mild impairments. This finding corroborates Asuman, Gerdtham, Alriksson‐Schmidt, Nordin, and Jarl (2024a), who argues that higher labor‐market participation among parents of children with severe impairments may have negative consequences for their mental health and quality of life.

It should be noted that we compared parents of children with CP to the general population that includes parents of children with other disabilities. This approach enables us to account for the fact the persons with CP may have other disabilities that are not caused by the underlying brain damage that resulted in CP. In addition to the motor impairment, persons with CP may experience intellectual (Reid et al. 2018) and neuro‐psychiatric (Påhlman et al. 2019) disabilities. This approach, we believe, enables us to minimize the risk of estimating biased effect sizes of the parental mental‐health consequences of having a child with CP. Further, our results show that comparing parents of children with CP to the general population is a policy‐relevant question, as parents of children with CP may be at a higher risk of poor mental‐health outcomes compared to the general population.

Our findings are in line with previous studies that have reported deteriorating mental‐health effects of parenthood in general (Ahammer et al. 2023; Hart et al. 2023; Dehos et al. 2024), child health shocks (Breivik and Costa‐Ramón 2024) and parenting children with disabilities specifically (Adhvaryu et al. 2023; Chen et al. 2023; Cheung et al. 2025). These papers have shown that parental mental penalties are more pronounced for mothers than fathers. This study, however, departs from the previous literature in a significant way. Unlike most previous studies that examined a single indicator of mental health, such as depression and the use of mental healthcare services, this paper examined a broad spectrum of mental‐health disorders. Our results show that fathers and mothers indeed face different mental‐health consequences due to having a child with a severe health condition. Thus, the use of single indicators of mental health may overlook the differences in the mental‐health effects of parenthood between fathers and mothers. In addition, CP is a permanent disability; as such, the negative mental health consequences may be prolonged compared to transitory health shocks such as hospitalizations (Breivik and Costa‐Ramón 2024).

Our results further highlight that in high‐income countries with comprehensive social welfare benefits and programs and family‐friendly policies, having a child with CP may constitute a significant mental health toll on parents. The mental‐health consequences of having a child with CP may, therefore, be larger in countries without comprehensive interventions that aim to support and assist children and parents in vulnerable situations. Further, the financial demands, including the medical expenses in settings with large user fees/co‐payments, of caring for a child with CP may contribute additionally to the pressure on parents, leading to a deterioration of their mental health (Selenko and Batinic 2011; Hiilamo 2020; Ryu and Fan 2023).

In Sweden, parents of children with disabilities may qualify for benefits from the welfare system, in addition to access to healthcare under a universally funded system. A key support system is personal assistance for the child with CP, where support is based on need, which is closely correlated to severity (von Granitz et al. 2017). However, the Swedish social welfare system has witnessed a shortage of personal assistants (Ahlström and Wadensten 2011), which has resulted in some children with disabilities not having access to the support they need. As a result, some parents may apply to take on the role of personal assistant in addition to the parental caregiving roles. Lichtenberg (2024) also finds an emergence of inequalities in access to personal assistance by parental educational attainment in Sweden over the period. Our results, however, do suggest that dispensed anti‐depression medications are higher for mothers with higher education than those with lower education.

There are some limitations to our analyses that must be highlighted. Mental health is a multifaceted phenomenon that goes beyond clinical diagnoses and dispensed medications. Our approach of using data on dispensed medications from administrative registers to measure parental mental health overcomes biases arising from recall or under‐reporting of self‐reported symptoms. It also has the added benefit of capturing the period when a person is on pharmacological treatment, as opposed to information on diagnoses. However, dispensed‐medication data does not capture individuals with mental conditions that are not on pharmacological treatment or have not sought healthcare. The effect sizes may, therefore, be underestimated if the likelihood of seeking treatment for mental‐health conditions differs between parents with and without a child with CP. Our findings suggest that the healthcare‐seeking behaviors of parents do not differ by the CP status of the child, indicating that such potential biases may not arise in this case. On the other hand, our measure of mental health may be seen as capturing persons with severe conditions who sought treatment within the healthcare system. Thus, the generalizability of our findings may be limited to such persons.

The second limitation relates to our empirical approach. The event‐study approach rests on the assumption that the mental health of parents of children with and without CP was similar before the birth of the child. However, this assumption may not hold for some mental health outcomes. Mental health challenges may emerge during pregnancy due to biological and social factors, especially for mothers. The results of the multiple hypothesis testing do, however, suggest that differences in the mental health of parents prior to the birth of the child are random rather than systematic.

Our findings have important policy implications, highlighting the need for support and assistance to families of children with disabilities. Poor parental mental health has been shown to affect cognitive, emotional, social, and behavioral development of children (Smith 2004; Mensah and Kiernan 2010; Manning and Gregoire 2009). In addition, studies have shown an inter‐generational persistence of poor mental health, such that children of parents with poor mental health are more likely to be diagnosed with a mental health disorder in later life (Bütikofer et al. 2024). A growing body of evidence shows negative consequences of poor mental health, including lower educational attainment (H. Shen et al. 2016; Ranning et al. 2018; Paget et al. 2018) and labor‐market outcomes (Banerjee et al. 2017; Mousteri et al. 2019; Y. Shen 2023; Wang et al. 2023). Thus, it is plausible that part of the excess mental health problems noted among persons with CP (Linder et al. 2024) and poorer outcomes in, for example, the labor market (Asuman, Gerdtham, Alriksson‐Schmidt, Nordin, and Jarl 2024b) may be due to the mental health strain on parents from having a child with CP, as noted in the current study.

Our results highlight that interventions targeted at new parents to cope with the stress and demands of parenting a child with CP may provide substantial public health and welfare gains. Particularly, increasing access to mental‐health services through incorporating parental mental‐health assessments during child healthcare visits may be crucial to effectively prevent and treat mental health conditions among parents of children with disabilities. Also, there is a need to invest in social support systems, such as personal assistants, for parents of children with disabilities to meet the care demands of their children.

While our findings underscore a pressing need for targeted interventions to mitigate the mental health burdens faced by parents of children with disabilities, it is crucial to recognize that identifying a problem does not inherently justify broad calls for intervention without rigorous evaluation. Effective policy measures require a thorough assessment, not only of their efficacy, but also of their cost‐effectiveness. Future research should focus on evaluating the impacts of specific interventions in real‐world settings to ensure that they provide meaningful benefits relative to their costs. Such evaluations are vital to ensure that limited resources are allocated in a manner that maximizes societal welfare.

## Conflicts of Interest

The authors declare no conflicts of interest.

## Figures and Tables

**FIGURE A1:**
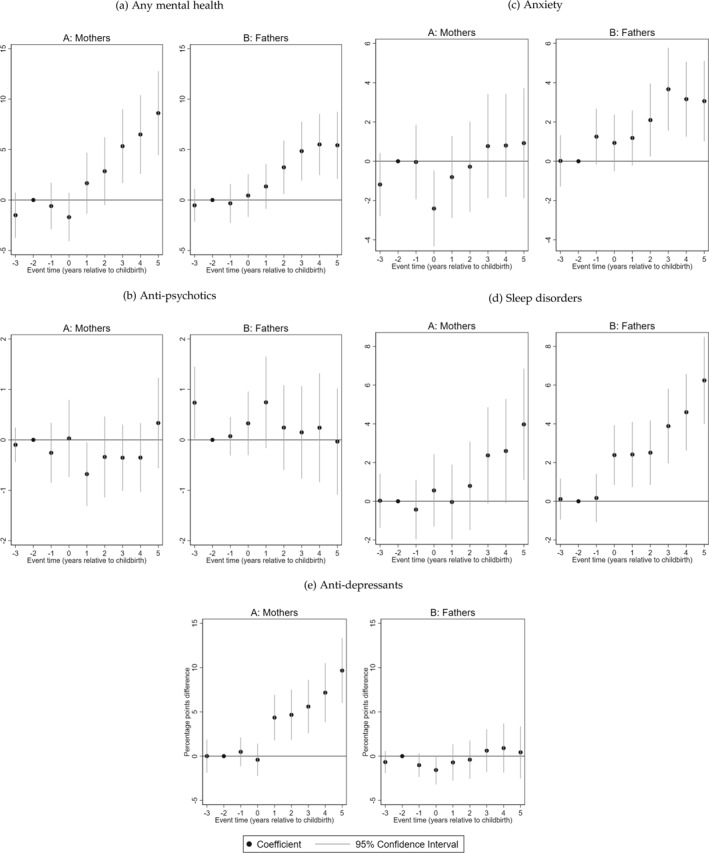
Total effect of having a child with CP on parental mental health. Event study estimates for mothers (Panel A) and fathers (Panel B). The outcomes are dispensed medication for various mental‐health disorders. The plot shows the total effect of having a child with CP compared to having a child without CP estimated by Equation (1). Spikes show the 95% confidence intervals. The regression includes controls for event‐time dummies, calendar year, and age dummies.

## Data Availability

Deidentified individual participant data will not be made available by the authors but are available from the register holders after typical application procedures.

## Endnotes

^1^
We clean the data extensively by excluding persons whose diagnosis of CP may be incompatible with the definition of CP. We exclude individuals who do not have a CP diagnosis after age four. Individuals with acquired brain damage after the age of two but no CP diagnosis before the brain damage are also excluded. Individuals are also excluded if they have other diagnoses that are considered incompatible with CP (ICD10: G60, G61, G62, G71, G72, G834, G95, E71, E72, E74, E75, E76, E830, G114, G12, G31, G37, Q06, Q743). Finally, for the current study, we exclude individuals not included in the CPUP register.

^2^
We exclude siblings from the control group.

^3^
We exclude children with spina bifida from the control group.

^4^
The total effects of having a child with CP are presented in Figure A1 in the Appendix A.

^5^
The median age at birth in our sample was 29 years for mothers and 31 years for fathers.
